# The anti-inflammatory agent bindarit acts as a modulator of fatty acid-binding protein 4 in human monocytic cells

**DOI:** 10.1038/s41598-019-51691-y

**Published:** 2019-10-22

**Authors:** Sergio Oddi, Lucia Scipioni, Antonio Totaro, Clotilde Angelucci, Beatrice Dufrusine, Annalaura Sabatucci, Daniel Tortolani, Isabella Coletta, Maria Alessandra Alisi, Lorenzo Polenzani, Michael Assfalg, Carlo Caltagirone, Enrico Dainese, Mauro Maccarrone

**Affiliations:** 1 Faculty of Veterinary Medicine, University of Teramo, Teramo, 64100 Italy; 2 European Center for Brain Research (CERC)/Santa Lucia Foundation IRCCS, Rome, 00143 Italy; 3 Angelini RR&D (Research, Regulatory & Development), Angelini S.p.A., Rome, 00181 Italy; 4 Department of Biotechnology, University of Verona, Verona, 37129 Italy; 5 Department of Medicine, Campus Bio-Medico University of Rome, Rome, 00128 Italy

**Keywords:** Mechanism of action, Monocytes and macrophages, Chemokines, Lipid signalling

## Abstract

We investigated the cellular and molecular mechanisms by which bindarit, a small indazolic derivative with prominent anti-inflammatory effects, exerts its immunoregulatory activity in lipopolysaccharide (LPS) stimulated human monocytic cells. We found that bindarit differentially regulates the release of interleukin-8 (IL-8) and monocyte chemoattractant protein-1 (MCP-1), enhancing the release of IL-8 and reducing that of MCP-1. These effects specifically required a functional interaction between bindarit and fatty acid binding protein 4 (FABP4), a lipid chaperone that couples intracellular lipid mediators to their biological targets and signaling pathways. We further demonstrated that bindarit can directly interact with FABP4 by increasing its expression and nuclear localization, thus impacting on peroxisome proliferator-activated receptor γ (PPARγ) and LPS-dependent kinase signaling. Taken together, these findings suggest a potential key-role of FABP4 in the immunomodulatory activity of bindarit, and extend the spectrum of its possible therapeutic applications to FABP4 modulation.

## Introduction

Bindarit [2-[(1-benzylindazol-3-yl)methoxy]-2-methylpropanoic acid] is a synthetic indazolic derivative with prominent anti-inflammatory activity, that has been shown to be safe and particularly effective in the treatment of several experimental inflammatory and autoimmune disorders, from viral and adjuvant arthritis to acute pancreatitis, diabetic nephritis and autoimmune encephalomyelitis^1–5^. Such a spectrum of activities has been associated with the ability of bindarit to inhibit the production of a defined set of related C-C motif chemokines, primarily MCP-1, and thus to interfere with monocyte recruitment and differentiation^1,6,7^.

A previous study has identified a modulatory effect of bindarit on the nuclear factor-κB (NF-κB) signaling pathway in a macrophagic cell line, where bindarit specifically inhibited p65 and p65/p50-mediated MCP-1 promoter activation^7^. However, the mechanistic details of this action remained unexplored.

Here, we used a human myeloid MonoMac-6 (MM-6) cells to interrogate the molecular and cellular mechanisms underlying the immunomodulatory activity of bindarit on the expression of LPS-induced MCP-1 and IL-8. In particular, by complementing *in silico*, biochemical and imaging approaches, we assessed the possible involvement of FABP4 and PPARγ. Indeed, cross-talks between these two proteins have been implicated in bioactive lipid signaling and in particular in regulating the expression of IL-8 and MCP-1 in monocytes and macrophages^8–12^.

## Results

### Bindarit differentially regulates LPS-mediated secretion of IL-8 and MCP-1

**Figure 1:**
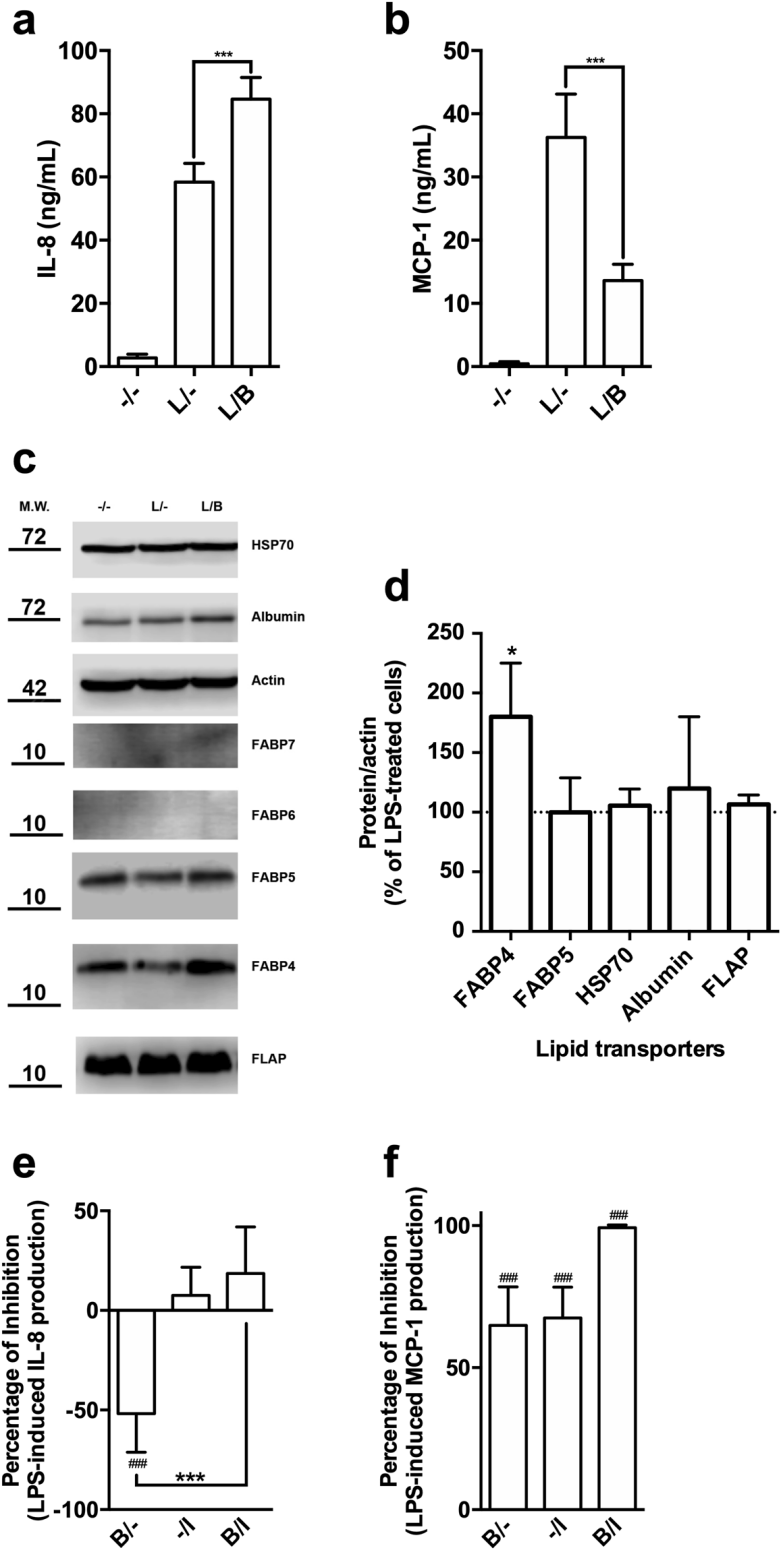
Study of the immuno-modulatory activity of bindarit on LPS-stimulated monocytes. MM-6 cells were left untreated (−/−) or were treated with 100 ng/mL LPS (L) for 20 hours, in the absence (L/−) or presence of 300 μM bindarit (L/B). After treatment, IL-8 (**a**) and MCP-1 (**b**) levels were measured in cell supernatants by means of AlphaLISA. Values are means ± S.D. of 5 independent experiments, each performed in duplicate. Significance is shown as *P* value, calculated with an unpaired *t*-test. ***P < 0.001. (**c**) Representative immunoblot showing the expression profile of different lipid binding proteins in MM-6 cells left untreated (−/−) or treated with 100 ng/mL LPS (L) for 20 hours, in the absence (L/−) or presence of 300 μM bindarit (L/B). Protein lysates were subjected to immunoblotting following 10% SDS-PAGE against the antibody indicated on the right side. Actin was also probed with anti-actin antibody to confirm equal protein loading. Molecular weights (M.W.) of protein markers (in kDa) are shown on the left side. The original scans were manipulated to remove irrelevant lanes and/or background regions. Full-length and original blots are presented in supplementary information. (**d**) Bar graph of the densitometric analysis of 5 independent experiments. The results were normalized with respect to the actin signal and reported as percentage of increase (±S.D.) with respect to the normalized value of the treatment with LPS. Significance is shown as *P* value calculated using a one-sample *t*-test. ^*^P < 0.05 *vs* LPS (set to 100%). Inhibition of the release of IL-8 (**e**) and MCP-1 (**f**) from LPS-stimulated monocytes by bindarit (B, 300 μM) and BMS309403 (I, 5 μM), used alone (B/− and −/I) or in combination (B/I). After treatment, chemokine content was analyzed in the supernatants by AlphaLISA and was expressed as percentage of inhibition of LPS-stimulated cells. Values are means ± S.D. of 5 independent experiments, each performed in duplicate. Significance is shown as *P* value, calculated using an unpaired *t*-test. ^###^*P* < 0.001 *vs* LPS (set to 0%). ***P < 0.001.

To explore the molecular mechanisms behind the anti-inflammatory activity of bindarit, we investigated the effect of this substance on the expression of MCP-1 and IL-8, two main pro-inflammatory chemoattractant proteins that are known to be targeted by bindarit^1,6,7^. Bindarit has already been used to inhibit the expression of MCP-1 in MM-6 cells at a concentration of 300 μM and with a treatment of 20 hours^1,6^. In preliminary experiments performed on these cells, we also made concentration-response curve of bindarit for the inhibition of MCP-1, finding that the IC50 of bindarit was 250 ± 60 μM (supplementary Fig. 1), very close to that reported from others^6^. Therefore, we decided to use bindarit at the concentration of 300 μM and for a 20-hour treatment to better amplify the modulatory effects of bindarit on the expression of the two cytokines of interest. In unstimulated cells, bindarit alone did not change the basal expression of the two cytokines (data not shown). Stimulation of MM-6 with LPS caused a robust increase of both MCP-1 and IL-8 levels compared with unstimulated cells (Fig. 1). Unexpectedly, pretreatment with bindarit differentially impacted on the expression of the two chemokines, increasing of ∼1.5-fold the production of IL-8 (*P* < 0.001) while inhibiting of ∼3-fold that of MCP-1 (*P* < 0.001; Fig. 1a,b).

### Bindarit selectively up-regulates the expression of FABP4

A likely molecular target of bindarit appears to be FABP4, a small cytosolic protein responsible for the intracellular transport of fatty acids in both adipocytes and macrophages, where it is a major regulator for the expression of MCP-1^8,9,13,14^. We hypothesized that bindarit could interfere with MCP-1 production by functionally inhibiting the activity of FABP4. To test this hypothesis, we firstly assessed the effect of bindarit on the expression of FABP4. LPS *per se* did not change the expression of FABP4, nor that of other carriers that were analysed (Fig. 1c). Unexpectedly, bindarit was found to induce a significant increase of FABP4 levels in LPS-stimulated monocytic cells (Fig. 1c,d). Notably, this effect was specific for FABP4, because bindarit did not affect the expression of FABP5, another member of FABP family that is expressed in monocytes^15^, nor that of other proteins involved in the intracellular transport of lipids in monocytes/macrophages, like albumin^16^, 70-kDa heat shock protein (Hsp70)^17^ and 5-lipoxygenase activating protein (FLAP)^18^ (Fig. 1c,d).

### FABP4 is involved in the mechanism of action of bindarit

A further investigation of the role of FABP4 on the immuno-modulatory activity of bindarit was carried out with BMS309403, a potent and selective inhibitor of FABP4^19^. BMS309403 *per se* failed to alter LPS-induced release of IL-8, while it completely reverted the bindarit-mediated over-expression of IL-8 (Fig. 1e; *P* < 0.01). In addition, BMS309403 alone robustly reduced the LPS-mediated release of MCP-1, which was completely erased upon co-administration of BMS309403 with bindarit (Fig. 1f; *P* < 0.01).

### Bindarit physically interacts with FABP4

**Figure 2:**
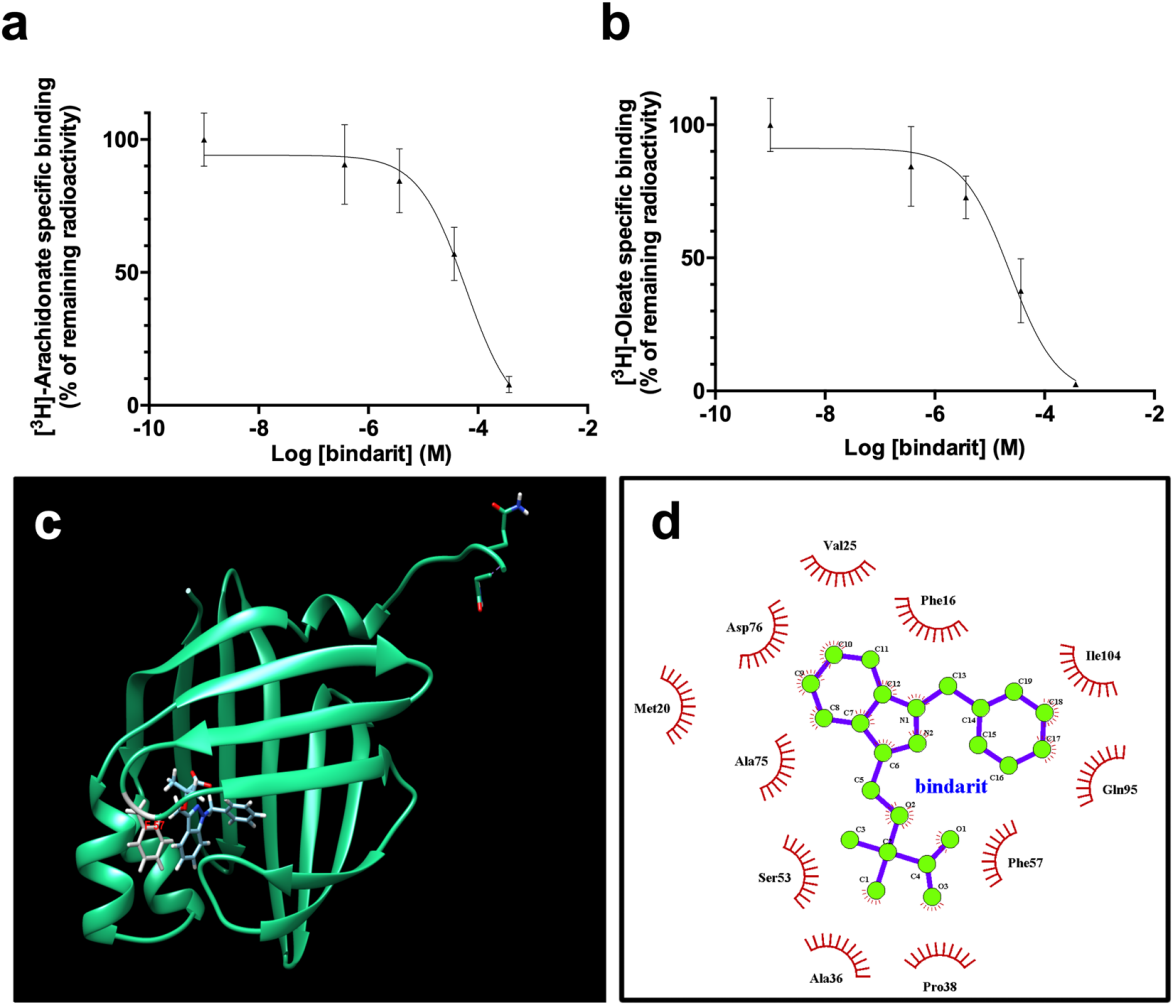
*In vitro* and *in silico* studies of the physical interaction between human FABP4 and bindarit. Displacement of [^3^H]-arachidonic (**a**) and [^3^H]-oleic acid (**b**) from the binding site of human FABP4 by bindarit. Displacement curves were fit to a one-site model with Ki values of 19 μM and 60 μM for arachidonate and oleate, respectively. The graphed points represent the means ± S.D. of 2 independent experiments, each performed in triplicate. (**c**) Bindarit has a binding mode similar to that of ibuprofen in the active site of human FABP4. Grey: residue Phe57, involved in the binding of small molecules. (**d**) 2D plot representation of the bindarit’s interactions with amino acid residues in the fatty acid binding pocket of the human FABP4.

To ascertain the possible physical between bindarit and FABP4, competitive binding assays were performed, in which increasing concentrations of bindarit were used to displace radiolabeled fatty acids bound to FABP4. Bindarit was shown to be able to fully displace both [^3^H]-oleic acid and [^3^H]-arachidonic acid (Fig. 2a,b), with Ki values of 19 ± 3 μM and 60 ± 17 μM, respectively.

To further investigate the possible association between bindarit and FABP4, a docking analysis was performed on the crystal structure of human FABP4 (pdb code 3p6g.pdb) with bindarit (Fig. 2c), and calculated binding energies and contacts were compared with those of the co-crystallized molecule ibuprofen. In its best binding mode bindarit was docked to the active site of FABP4 in a very similar conformation compared to ibuprofen. Consistently with these data, fullfitness values of −885 kcal/mol and −962 kcal/mol, and binding free energies of −8.1 kcal/mol and −6.9 kcal/mol, were obtained for bindarit and ibuprofen, respectively. Of note, residues in the ligand binding pocket involved in the binding of both compounds included Phe57 and Phe16 (Fig. 2d), which have been shown to make hydrophobic interactions with fatty acids and other small molecule inhibitors^20,21^. Altogether, these results strongly suggest that bindarit effectively binds to FABP4, more likely to the fatty acid binding site.

### Bindarit promotes nuclear import of FABP4

By *in silico* analysis it was predicted that bindarit binds to a region of FABP4 that is involved in the regulation of the nucleo-cytoplasmic distribution of the protein^20^. Indeed, it has been proposed that the binding of specific ligands to this regulation site induces intramolecular rearrangements that lead to the exposure of an otherwise hidden nuclear localization sequence, which enables FABP4 translocation from the cytosol into the nucleus^20,22^.

**Figure 3:**
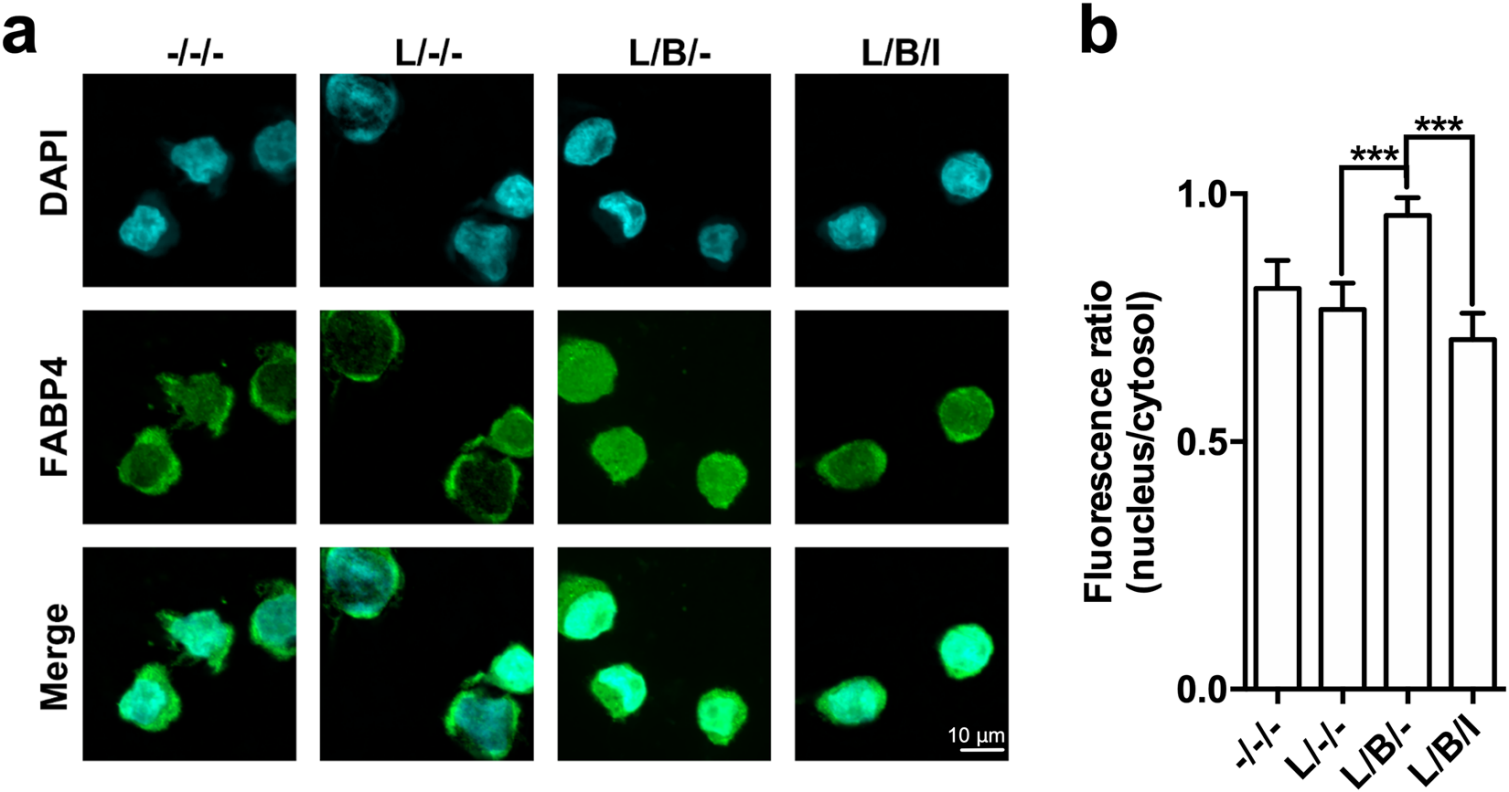
Effect of bindarit on the subcellular localization of FABP4 in MM-6 cells. (**a**) Indirect immunostaining of FABP4 in human monocytes. FABP4 was visualized by confocal immunofluorescence microscopy in MM-6 cells left untreated (−/−/−) or treated with 100 ng/mL LPS (L) for 20 hours, in the absence (L/−/−) or presence of 300 μM bindarit used alone (L/B/−) or in combination with 5 μM of BMS309403 (L/B/I). After each treatment, cells were fixed and stained with anti-FABP4 (green, middle panel) and with DAPI for nuclear labeling (cyan, top panels). Merged images are shown in the bottom panels. Images are representative of 2 independent experiments. (**b)** Ratios of fluorescence intensity between nucleus and cytosol of 20–28 cells under each condition were calculated as described in Materials and Methods, and are reported as mean ± S.D. Significance is shown as *P* value calculated using an unpaired *t*-test. ***P < 0.001.

In order to ascertain whether bindarit could promote nuclear translocation of FABP4 endogenous FABP4 was imaged in MM-6 cells by indirect immunofluorescence microscopy (Fig. 3a). The evaluation of the extent of nuclear translocation of FABP4 was performed by measuring the ratio of nuclear to cytoplasmic fluorescence of the protein. LPS-stimulated MM-6 cells displayed a similar subcellular localization of FABP4 compared to untreated controls (Fig. 3a). Instead, bindarit led to a marked increase in nuclear localization of FABP4 compared with LPS treatment (Fig. 3b; *P* < 0.001), supporting a role for bindarit as a nuclear-targeting ligand of the protein. In addition, BMS309403 blocked the bindarit-induced nuclear import of FABP4 (Fig. 3b; *P* < 0.001), suggesting that translocation of FABP4 into the nucleus is necessary for the immunoregulatory activity of bindarit.

### PPARγ activity is required for bindarit-mediated overexpression of FABP4 and IL-8

**Figure 4:**
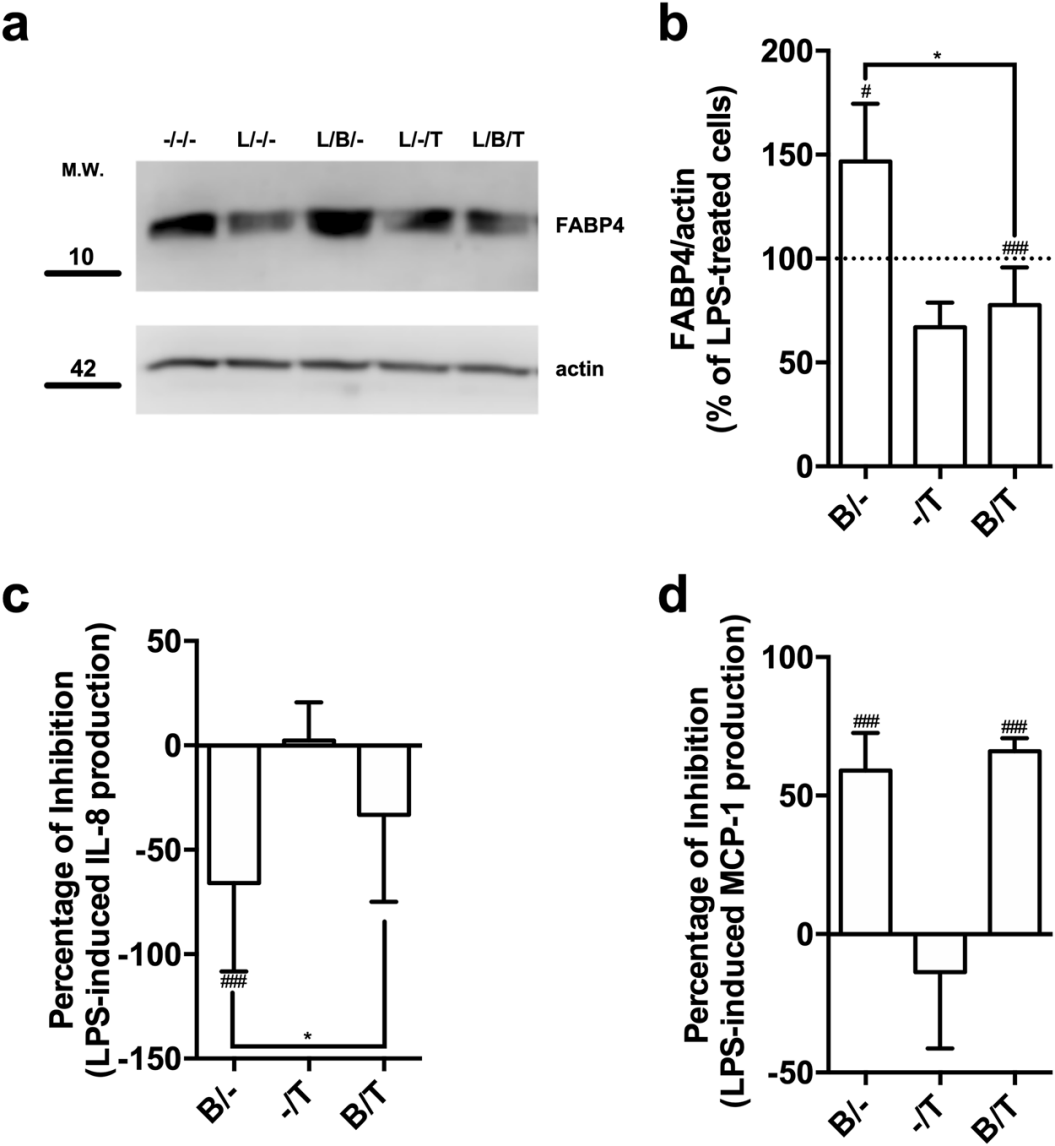
Effect of pharmacological inhibition of PPARγ on the immuno-modulatory activity of bindarit. (**a**) Representative immunoblot showing the expression FABP4 in MM-6 cells left untreated (−/−) or treated with 100 ng/mL LPS (L) for 20 hours, in the absence (L/−) or presence of 300 μM bindarit (B) and 2 μM T0070907 (T), used alone or in combination (L/B− an L/B/T). Protein lysates were subjected to immunoblotting following 10% SDS-PAGE against the anti-FABP4 antibody. Actin was also probed with anti-actin antibody to confirm equal protein loading. Molecular weights (M.W.) of protein markers (in kDa) are shown on the left side. (**b**) Bar graph of the densitometric analysis of 5 independent experiments. The results were normalized with respect to the actin signal, and were reported as percentage of increase (±S.D.) with respect to the normalized value of the treatment with LPS. Significance is shown as *P* value calculated using a one-sample *t*-test. ^#^*P* < 0.05 *vs* LPS (set to 100%). ^###^*P* < 0.001 *vs* LPS. *P < 0.05. Inhibition of the production of IL-8 (**c**) and MCP-1 **(d**) from LPS-stimulated monocytes by bindarit (B, 300 μM) and T0070907 (T, 5 μM), used alone (B/− and −/I) or in combination (B/I). After treatment, chemokine contents were analyzed in the supernatants by AlphaLISA and were expressed as percentage of inhibition of LPS-stimulated cells. Values are means ± S.D. of five independent experiments, each performed in duplicate. Significance is shown as *P* value calculated using an unpaired *t*-test. ^###^*P* < 0.001 *vs* LPS (set to 0%). *P < 0.05.

The increased expression of FABP4 induced in MM-6 cells by treatment with bindarit prompted us to investigate the involvement of PPARγ, a transcription factor known to control the expression of proteins involved in lipid transport/metabolism, FABP4 included^23–26^. Treatment with T0070907, a potent and selective PPARγ antagonist^27^, caused a drastic reduction in the expression of FABP4 compared to that of LPS-treated cells (Fig. 4a). Of note, PPARγ antagonism prevented bindarit from exerting its stimulatory effect on the expression of FABP4 (Fig. 4a,b). Moreover, the inhibition of PPARγ, that *per se* did not alter LPS-induced release of IL-8, significantly reduced bindarit-induced over-expression of IL-8 (Fig. 4c, *P* < 0.05). Instead, T0070907 did not affect the inhibitory action of bindarit on the release of MCP-1 from LPS-stimulated MM-6 cells (Fig. 4d).

### Bindarit increases the activation of p38α in LPS-stimulated MM-6 cells

**Figure 5:**
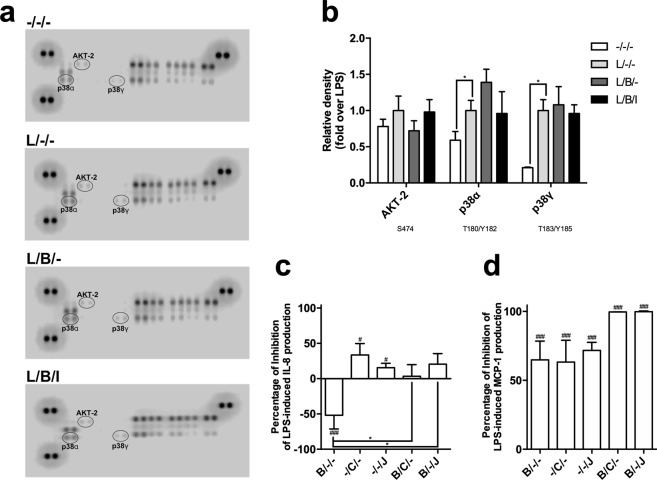
Impact of bindarit on LPS-induced activation of cellular kinases. MM-6 cells were left untreated (−/−/−) or treated with 100 ng/mL LPS (L) for 20 hours, in the absence (L/−/−) or presence of 300 μM bindarit (B), used alone (L/B/−) or in combination with 5 μM BMS309403 (L/B/I). Cell lysates containing 200 μg of total protein were analyzed for the relative levels of site-specific phosphorylation of selected kinases, by using the human phospho-mitogen-activated protein kinase array kit. (**a**) Representative images of phospho-kinase arrays for each treatment captured by C-DiGit blot scanner. Each kinase is spotted in duplicate. The pairs of dots in each corner (with the exception of the negative control pair at the lower left corner) are positive controls. Each pair of the most positive kinase dots is denoted by an ellipse, with the name of the corresponding kinases. (**b)** Graphical representation of the array signal intensities of AKT-2, p38α and p38γ. Below each kinase the specific phosphorylation site(s) detected is (are) reported. Relative spot phosphorylation was quantified by normalizing pixel density of the positive control to 100 and was expressed as a fraction (±S.D.) of the value of LPS-treated cells (n = 2). Significance is shown as *P* value calculated using a one-sample *t*-test. *P < 0.05. Effect of pharmacological inhibition of AKT-2 and p38α on the immuno-modulatory activity of bindarit. Inhibition of the release of IL-8 (**c**) and MCP-1 (**d**) from LPS-stimulated monocytes by bindarit (B, 300 μM), the AKT-2 inhibitor CCT128930 (C, 5 μM) and the p38α inhibitor JX-401 (J, 1 μM), used alone (B/−/−, −/C/− and −/−/J) or in combination (B/C/− and B/−/J). After treatment, chemokine content was measured in the supernatants by AlphaLISA and was expressed as percentage of inhibition of the values obtained in LPS-stimulated cells. Values are means ± S.D. of four independent experiments each performed in duplicate. Significance is shown as *P* value calculated using an unpaired *t*-test. ^#^*P* < 0.05 *vs* LPS (set to 0%). ^###^*P* < 0.001 *vs* LPS (set to 0%). *P < 0.05.

In order to identify the molecular pathway involved in the immunomodulatory activity of bindarit, impact of this substance was assessed on the phosphorylation profile of several kinases, along with their main protein targets, by using a kinase profiler array. Compared to controls, stimulation with LPS for 20 hours increased phosphorylation of p38α and p38γ (Fig. 5a,b; *P* < 0.05), and to a lower extent of AKT-2 (Fig. 5a,b). Pre-treatment with bindarit induced changes (yet not statistically significant) in the phosphorylation of p38α and AKT-2, which increased and decreased ∼1.4-fold, respectively, compared to LPS (Fig. 5a,b). To further ascertain the involvement of FABP4 in the mechanism of action of bindarit, we tested the effect of co-administration of this substance with BMS309403, and found that FABP4 inhibition fully reversed the changes in phosphorylation state of p38α and AKT-2 induced by bindarit (Fig. 5a,b).

The role of the latter two kinases in bindarit signaling was further interrogated by using JX-401 and CCT128930, two potent and selective inhibitors for p38α and AKT-2, respectively^28,29^. We found that JX-401 pretreatment of MM-6 cells inhibited over-expression of IL-8 induced by bindarit (Fig. 5c). Instead, inhibition of p38α *per se* markedly reduced the LPS-mediated secretion of both IL-8 and MCP-1, and its combination with bindarit completely blocked the release of MCP-1 from LPS-stimulated MM-6 cells (Fig. 5d, *P* < 0.01). Similarly, the inhibition of AKT-2 *per se* significantly reduced LPS-induced production of IL-8 and MCP-1 (Fig. 5c,d). In combination with bindarit, CCT128930 inhibited the over-expression of IL-8 induced by bindarit, and completely abolished the release of MCP-1 from LPS-stimulated MM-6 cells (Fig. 5d).

**Figure 6:**
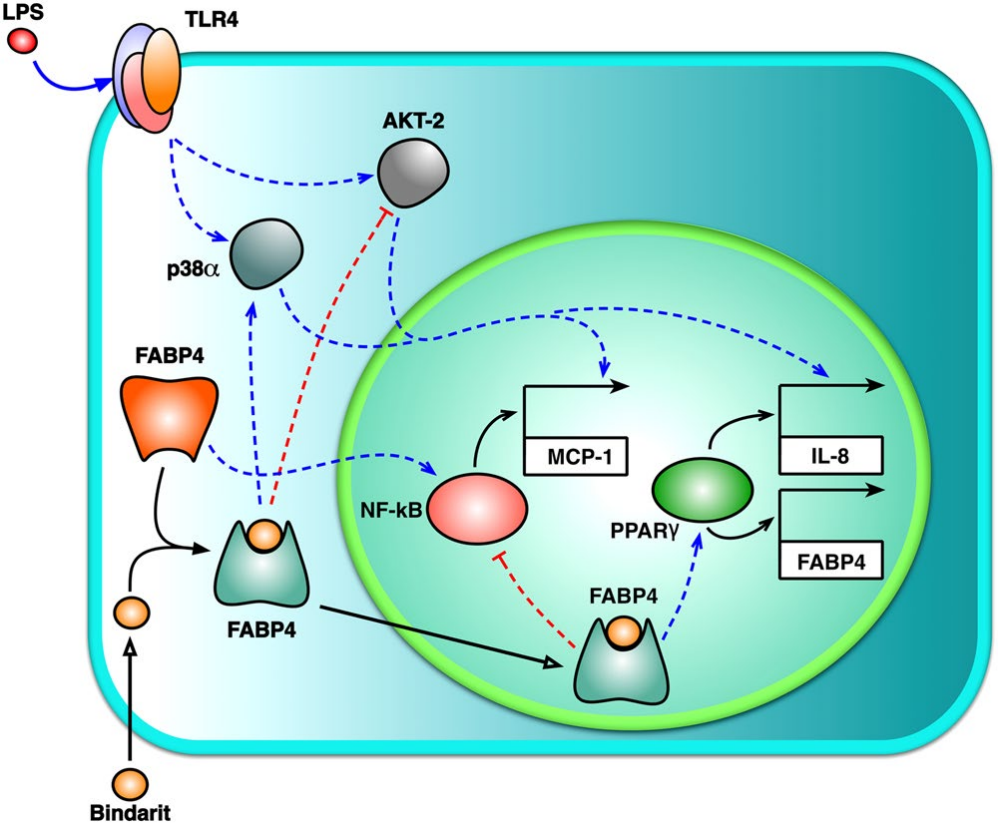
Proposed model for bindarit action. The potential mechanism of action of bindarit is presented. See text for details.

Altogether, these data demonstrated that both AKT-2 and p38α kinases are involved in the mechanism by which bindarit modulates pro-inflammatory signaling pathways triggered by LPS in human monocytes (Fig. 6).

## Discussion

In this study, we examined the cellular and molecular mechanisms underlying immunoregulatory activity of bindarit on the secretion of IL-8 and MCP-1 from LPS-stimulated human leukemic monocytes. We found that FABP4 is a key element in mediating the immunomodulatory effects of bindarit. In particular, we showed that inhibition of FABP4 reverts bindarit-mediated overexpression of IL-8, while potentiating downregulation of MCP-1. The involvement of FABP4 in the mechanism of action of bindarit was further corroborated by the findings that the latter substance: (*i*) specifically upregulates the expression of FABP4 via PPARγ, (*ii*) directly binds FABP4 with high affinity, and (*iii*) mediates its translocation into the nucleus, where (*iv*) it likely promotes PPARγ transcriptional activity. Finally, we observed that pre-treatment with bindarit alters the phosphorylation state of p38α and AKT-2 in a FABP4-dependent manner.

Bindarit exerts its anti-inflammatory activity by inhibiting the production of a defined set of chemokines, primarily MCP-1^1,6,7^. Here, we provided unprecedented evidence that, besides inhibition of MCP-1 production, bindarit can increase LPS-stimulated synthesis of IL-8 (Fig. 1), thus highlighting a more complex role of this small indazolic molecule in regulating inflammatory pathways.

We found that bindarit increased the expression of FABP4, but not of FABP5 nor of other lipid-binding proteins, in a PPARγ-dependent manner. Since PPARγ agonism has been reported to increase the expression of both FABP4 and IL-8^12,26,30^, it seems plausible that bindarit can activate this nuclear transcription factor. This possibility seems to be further supported by the fact that the pharmacological inhibition of PPARγ blocks stimulatory effects of bindarit on FABP4 and IL-8 expression (Fig. 4). In this context, it should also be mentioned that several FABP4 ligands, such as ibuprofen and arachidonic acid^31,32^, also behave as PPARγ agonists/ligands^33,34^. The high affinity of FABP4 for bindarit and the high similarity of its binding pocket to that of ibuprofen (Fig. 2) suggest that bindarit could indeed bind also to PPARγ in much the same way as ibuprofen. The observation that FABP4 massively relocates to the nucleus in the presence of bindarit (Fig. 3), thus delivering the drug in close proximity of nuclear PPARγ, seems to further support this concept. However, a possible physical and functional interaction between bindarit and PPARγ should be further investigated in an independent study.

LPS-induced MCP-1 and IL-8 expression in myeloid cells is mediated by Toll-like 4 receptor (TLR4), that triggers a very complex signaling, that engages p38, p44/42 and c-Jun N-terminal kinase (JNK) MAPKs, as well as phosphoinositide 3-kinase (PI3K)/AKT and other transcription factors like NF-κB^35^. Here, bindarit was found to alter — in an opposite way compared to LPS — the phosphorylation pattern for p38α and AKT-2 (Fig. 5). These results indicate that the two kinases, that have been shown to work together in LPS-induced overexpression of IL-8 and MCP-1^36–38^, are downstream effectors of the immunomodulatory activity of bindarit. Incidentally, the mechanism by which bindarit exerts its differential effects on the activation of p38α and AKT was not explored in detail, and remains to be ascertained in an independent investigation. At any rate, since FABP4 has been demonstrated to interact with Janus kinase 2 (JAK2)^39^ and phosphatase and tensin homolog (PTEN)^40^, which both regulate p38 and PI3K/AKT pathways^41,42^, it is conceivable that bindarit binding to FABP4 may alter phosphorylation of AKT-2 and p38α through JAK2 and PTEN.

A major outcome of this study is that bindarit can act as a modulator of FABP4 activity. The latter is a small molecular weight (~15 kDa) intracellular protein, primarily expressed in adipocytes and myeloid cells, where it noncovalently binds to: *(i)* several fatty acids, such as oleic acid and arachidonic acid^43,44^, (*ii*) eicosanoids, such as the leukotriene A4 (LTA4)^45^, and (*iii*) PPAR agonists, such as the troglitazone^46^. It should be recalled that FABP4 has been implicated in lipid sensing and immune response in monocytes/macrophages^15^. There is growing evidence that FABP4 could modulate inflammatory activity of these cells by acting at multiple levels such as: (*i*) delivering bioactive lipids to their molecular targets; (*ii*) sequestering lipid ligands, thus blocking their signaling cascades; (*iii*) favoring synthesis and signaling of major LTs, like LTB_4_, LTC_4_ and LTD_4_, through stabilization of their unstable precursors (*i.e*., LTA_4_). In its role as a lipid chaperone, FABP4 has been shown to promote the transcriptional activity of PPARγ by mediating the transfer of its lipophilic agonists from the cytoplasm into the nucleus, where it may then drive the ligand to its receptor target^22,46^. FABP4 has been also reported to attenuate anti-inflammatory lipid signaling by sequestering lipid mediators and preventing them from interacting with PPARγ and other targets, such as components of the IκB kinase (IKK)/NF-κB pathway^13,47,48^. Finally, FABP4 has a key role in the synthesis of LTs by prolonging the half-life of their unstable epoxide-containing precursor LTA_4_^45^. Accordingly, it has been reported that the genetic and pharmacological depletion of FABP4 in LPS-stimulated macrophages reduces production of LTC_4_^49^, as well as signaling cascades triggered by LTB_4_ and by its cognate receptor, BLT1^50^.

Keeping in mind the complex activity carried out by FABP4 in lipid signaling, as well as its direct functional interaction with PPARγ receptors, we can propose FABP4 as a key element in the mechanism of action of bindarit (Fig. 6). By interacting with FABP4 and modulating its (*i*) expression, (*ii*) binding of endogenous lipid ligands, and (*iii*) subcellular distribution, bindarit would be able to modulate PPARγ and hence LT metabolism and signaling thereof in LPS-stimulated monocytes. As for its activation of transcriptional activity of PPARγ, bindarit could have the double effect of stimulating the expression of IL-8^30^ and inhibiting that of MCP-1, possibly through the PPARγ-mediated antagonism of IKK/NF-κB pathway^13,47,48,51,52^. In the context of LT metabolism, it should be mentioned that LTs are potent inducers of MCP-1 and that stimulation of their receptors leads to MAPK- and PI3K/AKT-dependent activation of NF-κB^53,54^. A previous study demonstrated that indeed bindarit inhibits LPS-induced MCP-1 through a reduction in IκBα phosphorylation and nuclear translocation and transcriptional activity of NF-κB dimers^7^. Present results support the hypothesis that the inhibitory activity of bindarit on NF-κB pathway may be due, at least in part, to an interference with FABP4-dependent LT signaling pathways.

In conclusion, this investigation demonstrates that the immunomodulatory effects of bindarit depend on its capacity to selectively bind FABP4, thus functionally altering its activity and localization. Although further studies are deemed necessary to clarify how this interaction affects the distinct LPS-dependent signaling cascades, it is undoubted that the identification of an interplay between bindarit and FABP4 can open new perspectives for the exploitation of bindarit in the treatment of diseases where FABP4 plays a pivotal role, such as insulin resistance, type 2 diabetes, atherosclerosis and carcinogenesis^55^.

## Methods

### Reagents

Chemicals were of the purest analytical grade. Bindarit was provided by Angelini Research Center/ACRAF (Rome, Italy). RPMI 1640, fetal bovine serum (FBS), and other cell culture reagents were purchased from Corning (Corning, NY, USA). BMS309403 was from Cayman Chemical (Ann Arbor, MI, USA), JX-401 (Abcam, Cambridge, UK) and CCT128930 (Selleck Chem, Munich, Germany). Oleic acid, [9,10-^3^H(N)] (37 MBq), arachidonic acid [5,6,8,9,11,12,14,15-^3^H] (1850 KBq), Lipidex 1000 resin and Ultima Gold XR were purchased from PerkinElmer (Milan, Italy). All other chemicals were purchased from Sigma-Aldrich (St. Louis, MO, USA), unless stated otherwise.

### Cell culture and treatment

Human MM-6 cells were obtained from the Leibniz Institute DSMZ-German Collection of Microorganisms and Cell Cultures (Braunschweig, Germany; cat. no. ACC 124). MM-6 cells were cultivated in RPMI 1640 medium supplemented with 1% non-essential amino acids, 10 mg/mL sodium pyruvate, 10% FBS, 1% ng/mL human insulin and 1 nM oxaloacetic acid. The cells were grown without antibiotics at 37 °C in humidified atmosphere containing 5% CO_2_. The cells were maintained at the recommended density of 3 × 10^5^ cell/mL and used no later than 20^th^ passage. For cytokine expression analysis, cells (10^5^/well) were seeded into 96-well plates in 200 μL of RPMI 1640 medium (without serum) and were left untreated or treated for 1 hour with bindarit (300 μM), BMS309403 (5 μM), and CCT128930 (5 μM) and JX-401 (1 μM) used alone or in combination, and were then stimulated with 100 ng/mL of LPS (0111:B4; Sigma-Aldrich) for 20 hours. Stock solutions for bindarit, BMS309403, and CCT128930 and JX-401 were prepared in DMSO and diluted in free-FBS medium (final concentration of DMSO less than 0.1%). For Western blot and kinase profiler analyses, cells (10^6^/well) were seeded into 6-well plates in 2 mL of RPMI 1640 and treated as described above. Following incubation, cell suspensions were centrifuged at 3,000 × *g* for 10 minutes, and the cells and supernatants were removed and stored at −80 °C until they were used to measure protein and cytokine levels.

### Determination of cytokine secretion

The concentrations of human IL-8 and MCP-1 were determined by Amplified Luminescent Proximity Homogeneous Assay (Alpha)LISA using AL328C and AL244C kit, respectively, from PerkinElmer, according to manufacturer’s instructions. AlphaLISA is a bead-based technology which allow to detect analytes by homogeneous, no-wash immunoassay with high sensitivity and wide dynamic ranges^56^.

### Western blotting

Aliquots from MM-6 cells (50 μg/lane) were subjected to 10% SDS-PAGE under reducing conditions, then gels were electroblotted onto nitrocellulose filters (Whatman, Springfield Mill, UK) and immunoreacted with the following antibodies: mouse anti-actin (1:10000, Sigma Aldrich, cat. no. A-5441); rabbit anti-FABP4 (1:500, Abcam; cat. no. ab9250); rabbit anti-FABP5 (1:2000, Cell Signaling Technology, Danvers, MA, USA; cat. no. 39926); rabbit anti-HSP70 (1:1000, Cell Signaling Technology; cat. no. 4873); mouse anti-albumin (1:100, Cell Signaling Technology; cat. no. 4929) and rabbit anti-FLAP (1:500; Abcam, cat. no. ab85227). After incubation with the appropriate horseradish peroxidase-conjugated antibody (1:10000; Santa Cruz Biotechnology, Santa Cruz, CA, USA; sc-2004 and sc-2005), membranes were developed using an enhanced chemiluminescence detection system, according to the manufacturer’s instructions (Luminata Crescendo Western HRP substrate, Millipore, Burlington, MA, USA). Chemiluminescence signals were detected in a C-DiGit blot scanner (LI-COR, Lincoln, NE, USA) and analysed by Image Studio Software version 4.0.21 for Windows (LI-COR). Densities of protein bands in the Western blots were measured, and mean ratios between proteins and actin were reported.

### Assessment of the physical interaction between bindarit and FABP4

For ligand interaction assays, human FABP4 was recombinantly expressed in *E. coli*. Protein purification was carried out using established chromatographic procedures^57^. The purity of the sample was estimated using SDS-PAGE. To assess the ability of bindarit to bind FABP4, we performed displacement assays in which bindarit was used as a competitor of radiolabeled fatty acids ([H3]-arachidonic or [H3]-oleic acid) for binding to FABP4. Briefly, a fixed amount of human FABP4 (10 μg) was dissolved in 150 μL of a binding buffer (10 mM Tris-HCl, pH 8.0, 100 μM TX-100, 1 mM DTT) and incubated in 1.5-mL polypropylene tubes (Eppendorf, Hamburg, Germany) with 0.5 μM radioactive fatty acids in the absence or presence of growing concentrations of bindarit (final concentration of ethanol did not exceed 1% (v/v)). After incubation at 37 °C for 30 minutes, the solution was chilled on ice and mixed with 50 μL of a 50% (v/v) suspension of Lipidex 1000 resin, incubated on ice for 10 min with occasional stirring, and centrifuged for 2 minutes at 12,000 × *g* at 4 °C, to remove FABP4-unbound/displaced radioactive fatty acids. An aliquot of 100 μL supernatant was taken from each sample for counting FABP4-bound residual radioactivity in a PerkinElmer Tri-Carb 2810 TR scintillation counter. Blank samples (no added FABP4) were prepared for background subtraction for each condition. Displacement was monitored by decrease in net amount of radioactivity bound to FABP4. The inhibition constants (Ki) were determined by plotting the percentage of FABP4-remaining radioactivity against the bindarit concentration using nonlinear regression in GraphPad Prism version 6.00 for macOS (GraphPad Software, La Jolla, CA, USA).

### Docking analysis

The docking analysis was performed with the freeware online web service Swissdock based on the docking software EADock DSS (Dihedral Space Sampling), that allows prediction of the possible binding modes of a small molecule with a target protein, based on the CHARMM set of force fields. According to the software, the protein is mapped in a 3D grid, and cavities representing potential pockets are identified on the protein surface. The pockets with dimensions compatible with those of the ligand are chosen, and the dihedral angles of the ligand are optimized to obtain the ligand conformation that best fits the identified cavity. Then, a large number of binding modes (typically from 5,000 to 15,000) are generated for the ligand in the vicinity of the target cavity. Simultaneously, their CHARMM energies are estimated and energy minimization calculations are performed. Binding modes with the most favourable energies (ΔG) are ranked and clustered by RMSD with a distance cutoff of 2 Å. Finally, for each binding mode the software calculates the fullfitness value, an energetic parameter minimizing the target-ligand complex stability that takes into account different contributions, including the solvation energy for the complex. Ligand structures (inhibitors and fatty acids) were retrieved from the ZINC database. Visualization and analysis of molecular structures and related data were performed by means of the UCSF Chimera developed by the Resource for Biocomputing, Visualization, and Informatics at the University of California, San Francisco (supported by NIGMS P41-GM103311). Two-dimensional diagrams of ligand binding sites were drawn with the program Ligplot+.

### Immunofluorescence

After each treatment, MM-6 cells (10^5^ cells/mL) were plated on glass coverslips in 12-well plates and fixed with 3% paraformaldehyde containing 4% sucrose in PBS for 20 minutes. The cells were then washed with PBS and permeabilized for 10 minutes with PBS containing 5% bovine serum albumin, 0.1% NP-40 and 0.5% saponin. The immunodetection of FABP4 was carried out by incubating cells with rabbit anti-FABP4 primary antibody diluted 1:200 in PBS. After incubation with primary antibody for 3 hours, cells were washed and incubated for 1 hour at room temperature with Alexa Fluor 488-conjugate goat anti-rabbit secondary antibody (Molecular Probes, Eugene, OR, USA) diluted 1:200 in PBS. Cells were then DAPI counterstained, mounted with Prolong Gold Diamond (Molecular Probes) and imaged using a LSM 400 confocal microscope (Zeiss, Oberkochen, Germany), equipped with HCX plan apo 63X (numerical aperture 1.4) oil immersion objective. Green fluorescence was excited using a 488 nm laser line and the corresponding fluorescence emission was detected using a 580–620 nm bandpass filter, whereas DAPI was excited with a dedicated 405 nm UV diode, and emitted light filtered using spectral separation slits (415–490 nm). Each image was taken at the equatorial plan of the cells, using the ZEN software (Zeiss). For presentation purposes, images were exported in TIFF format and processed with Artstudio Pro version 2.0.13 for macOS (Lucky Clan, Lodz, Poland; http://www.luckyclan.com), for adjustments of brightness and contrast. For image analysis, data from high-resolution images of 15 cells from 2 independent experiments were acquired for each sample. Quantification of the nuclear and cytosolic mean fluorescence of FABP4 was carried out using ImageJ software (NIH, Bethesda, MD, USA), available at http://imagej.nih.gov/ij/.

### Phospho-MAPK antibody array

The profile or protein phosphorylation in untreated and treated cell lysate was conducted by using Human Phospho-MAPK Antibody Array (R&D Systems, Minneapolis, MN, USA; cat. no. ARY002) following the manufacturer’s instructions. Protein spots were visualized using the chemiluminescence detection reagents supplied in the kit. The intensity score of each duplicated array spot was captured by C-DiGit blot scanner and analysed by Image Studio Software, and the averaged intensity was calculated by subtracting the averaged background signal. The fold change was obtained by comparing samples with the LPS-treated sample (set to 1).

### Statistical analysis

Data reported in this paper are the means ± standard deviations (S.D.) of at least two independent experiments, each performed in duplicate or triplicate. *P* < 0.05 was chosen to establish significance. All measurements were plotted and analysed using GraphPad Prism version 6.00 for macOS (GraphPad Software).

## Supplementary information

Supplementary Information
