# Supplementary material for: The anti-inflammatory agent bindarit acts as a modulator of fatty acid-binding protein 4 in human monocytic cells
Source: Sci Rep. 2019 Oct 22;9:15155. doi: 10.1038/s41598-019-51691-y (PMC6805920; doi:10.1038/s41598-019-51691-y)

Supplementary Information

The anti-inflammatory agent bindarit acts as a modulator of fatty acid-binding protein 4 in human monocytic cells

Sergio Oddi[a,b], Lucia Scipioni[b], Antonio Totaro[b], Clotilde Angelucci[a], Beatrice Dufrusine[a], Annalaura Sabatucci[a], Daniel Tortolani[a], Isabella Coletta[c], Maria Alessandra Alisi[c], Lorenzo Polenzani[c], Michael Assfalg[d], Carlo Caltagirone[b], Enrico Dainese[a#] and Mauro Maccarrone[b,e#,*]*

Contents: Concentration-response curve of bindarit; full length blots of Figure 2 and Figure 6

Supplementary Figure 1

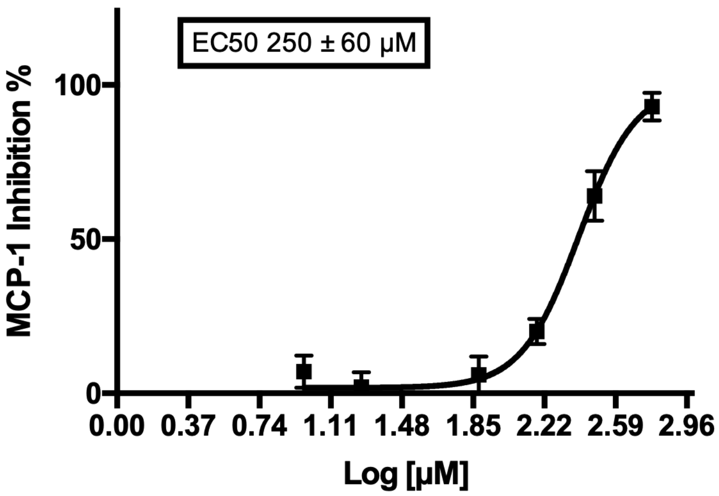

Supplementary Figure 1. Concentration-response curve of bindarit. Results are expressed as percent of inhibition and represent mean ± S.D. of 2 experiments.

Original blots for Figure 1

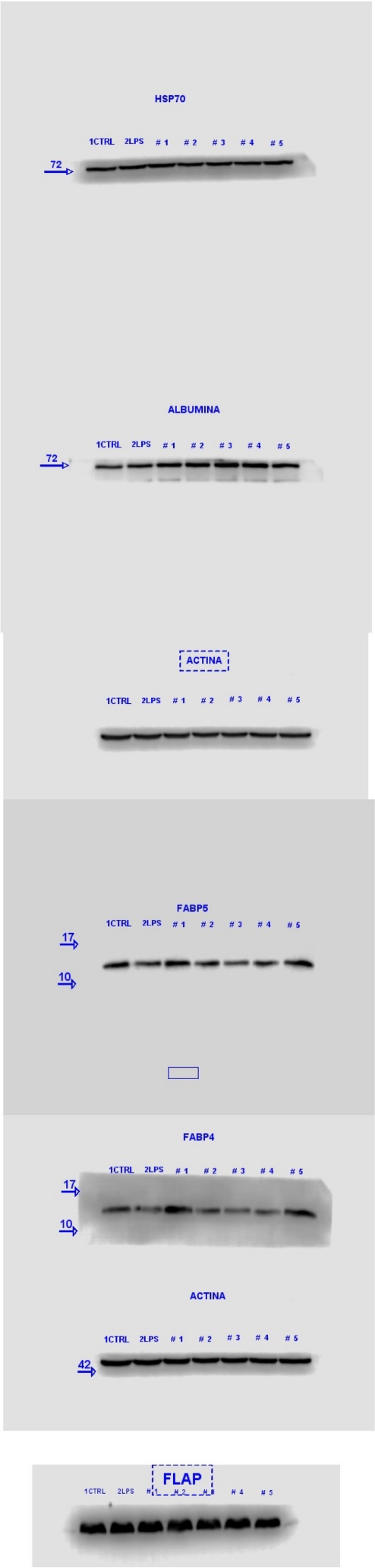

Original blots for Figure 4 for the immunodetection of FABP4 in MM-6 cells.

Lanes
1:CTRL
2: LPS
3: Compound #1: bindarit

Exp#31

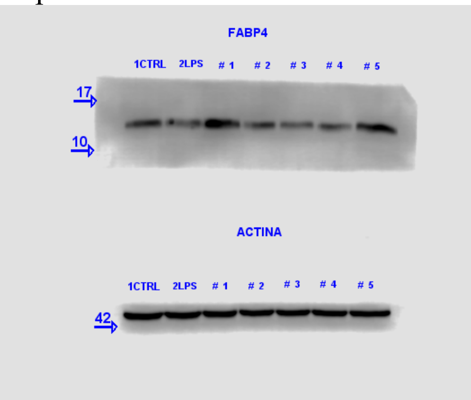

Exp#34

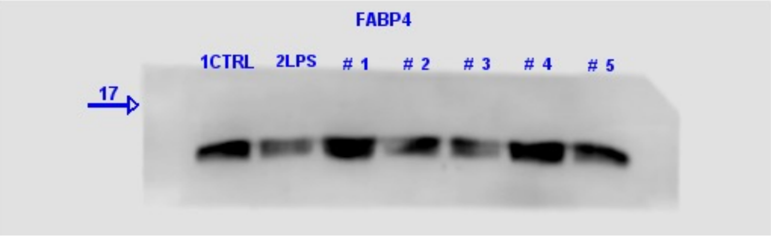

Exp#38

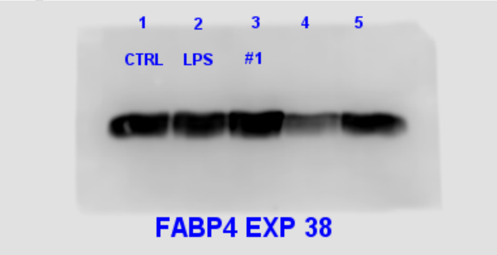

Exp#39

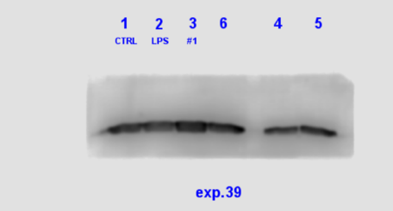

Exp#54

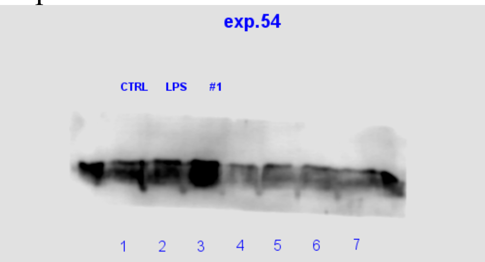

Supplement: Supplementary file 1 — Supplementary Information [file 41598_2019_51691_MOESM1_ESM.pdf]
